# Transcranial Irradiation Mitigates Paradoxical Sleep Deprivation Effect in an Age-Dependent Manner: Role of BDNF and GLP-1

**DOI:** 10.1007/s11064-023-04071-y

**Published:** 2023-12-20

**Authors:** Radwa H. Lutfy, Amina E. Essawy, Haitham S. Mohammed, Marwa M. Shakweer, Sherine Abdel Salam

**Affiliations:** 1 Department of Zoology, Faculty of Science, Alexandria University, Alexandria, 21511 Egypt; 2 School of Biotechnology, Badr University in Cairo, Badr City, Cairo 11829 Egypt; 3 Department of Biophysics, Faculty of Science, Cairo University, Giza, Egypt; 4 Department of Pathology, Faculty of Medicine, Badr University in Cairo (BUC), Cairo, Egypt; 5 Department of Pathology, Faculty of Medicine, Ain Shams University, Cairo, Egypt

**Keywords:** Paradoxical sleep deprivation, NIR PBM, Hippocampus, BDNF, GLP-1, Cell death

## Abstract

The growing prevalence of aged sleep-deprived nations is turning into a pandemic state. Acute sleep deprivation (SD) accompanies aging, changing the hippocampal cellular pattern, neurogenesis pathway expression, and aggravating cognitive deterioration. The present study investigated the ability of Near Infra Red (NIR) light laser to ameliorate cognitive impairment induced by SD in young and senile rats. Wistar rats ≤ 2 months (young) and ≥ 14 months (senile) were sleep-deprived for 72 h with or without transcranial administration of NIR laser of 830 nm. Our results showed that NIR photobiomodulation (PBM) attenuated cognitive deterioration made by SD in young, but not senile rats, while both sleep-deprived young and senile rats exhibited decreased anxiety (mania)-like behavior in response to PBM. NIR PBM had an inhibitory effect on AChE, enhanced the production of ACh, attenuated ROS, and regulated cell apoptosis factors such as Bax and Bcl-2. NIR increased mRNA expression of BDNF and GLP-1 in senile rats, thus facilitating neuronal survival and differentiation. The present findings also revealed that age exerts an additive factor to the cellular assaults produced by SD where hippocampal damages made in 2-month rats were less severe than those of the aged one. In conclusion, NIR PBM seems to promote cellular longevity of senile hippocampal cells by combating ROS, elevating neurotrophic factors, thus improving cognitive performance. The present findings provide NIR as a possible candidate for hippocampal neuronal insults accompanying aging and SD.

**Supplementary Information:**

The online version contains supplementary material available at 10.1007/s11064-023-04071-y.

## Introduction

Sleep is a universally conserved process within the animal kingdom [1]. Indeed, sleep is not a passive state, but a heavily active process that plays a role in clearance of buildup protein, and upregulation of many anabolic hormones, such as growth hormone and prolactin [2]. Growing evidence shows that sleep is regulated by different cortical and subcortical regions, such as the medial prefrontal cortex, amygdala, and hippocampus [3]. Furthermore, hippocampal memory consolidation was proven to occur during sleep, especially during slow wave sleep and rapid eye movement (REM) sleep [4], where minimal cholinergic neurons, which prevent hippocampus hypoactivity, lead to the consolidation of memories through the redistribution of new memories into the neocortex [5]. The role of sleep in high cognitive function is essential, where the lack of sleep effect has been phylogenetically conserved within different animal species [6]. Sleep deprivation (SD) was proven to impact long-term memory retention [7], hinder spatial working memory [8], and severely reduce the ability to discriminate between fear-relevant and safety cues [9].

It has been suggested that the hippocampus is particularly vulnerable to even little as 5–6 h of total SD, leading to a further reduction of spine density in the CA1, but not in CA3 subregion [10]. In addition, chronic short sleep restriction was reported to reduce CA1 neuron counts and volume, and CA1 glial activation [11]. The lack of sleep was reported to inhibit cellular proliferation of granular cells in the Dentate Gyrus (DG) and CA1 pyramidal neurons of the hippocampus [12]. Other published data have indicated that aging significantly disrupts sleep-dependent memory consolidation and contributes to age-related hippocampal dysfunction [13]. It was shown that age-related cholinergic hypofunction has repercussions on procedural memory consolidation taking part in REM sleep [14].

Previous studies conducted in animal models showed that both aging and SD have the ability to initiate oxidative stress, as reflected by elevated lipid peroxidation procucts (e.g., malondialdehyde) and depleted antioxidant defense in the hippocampus [15, 16]. The skyrocketing Reactive Oxygen Species (ROS) contribute to the hippocampal neuron damage by propagating pro-apoptotic protein, triggering apoptosis [17], and reducing the mature neurotrophic factors such as brain-derived neurotrophic factor (BDNF) [13]. BDNF has been known to play a pivotal role in SD, where a reduction in BDNF was reported in the case of SD, causing a substantial loss of adult born-neurons, increasing anxiety-like behavior in animals [9], and decreasing long-term potentiation [18]. Furthermore, SD was proven to exacerbate cellular injuries induced by six hydroxydopamine-lesioned rats by exercising an apoptotic effect, altering the Bax and Bcl-2 apoptotic genes and reducing BDNF [19]. This leads to reduced hippocampal neurogenesis, volume, loss of neurons, and cognitive decline [20]. Furthermore, cellular aging was found to reduce Glucagon-like peptide 1(GLP-1) [21], a peptide hormone identified as a gastrointestinal hormone but recently known for its role in stress adaption and regulation mechanisms [22]. GLP-1 dysfunctionality exerts a crucial effect in the development of neurodegenerative disease [23]. GLP-1 has gained attention due to its role in growth regulation, neuronal survival against neuroinflammation, amyloidogenesis, and cerebral glucose deprivation [24].

There is a need to find new treatment for SD with limited or no side effects and minimal invasive ability. Literature points to the near-infrared (NIR) laser as a potential neuroprotective agent that can influence neuronal status [25]. NIR transcranial treatment, also known as photobiomodulation (PBM) therapy, uses non-ionizing light sources such as lasers, LEDs, and broadband light in the visible and infrared range emitting NIR light (wavelengths between 800 and 2500 nm). If an optical window between 650 and 1200 nm is employed, the produced light can pass through the physical barriers of the skin and skull and reach the brain parenchyma [26]. It is a nonthermal process in which endogenous chromophores cause events at different biological scales. It has been proven to promote metabolic pathways that support positive therapeutic effects, such as reducing pain or inflammation, immunomodulation, and stimulation of tissue healing [27].

Several studies have pointed to the neuroprotective ability of transcranial application of 830 nm NIR against neurodegenerative disease, such as traumatic brain injuries, strokes, and major depression disorders [28]. In addition, NIR has been demonstrated to increase cell survivival, decrease ß-amyloid aggegates and improve behavioral state in animal models of Alzheimer’s disease [29]. Other researchers reported that NIR may be used to raise aged brain and behavioral functions by promoting oxidative metabolic capacity of neurons [30]. NIR PBM was confirmed to boost endogenous antioxidants, prevent apoptotic effect, and improve sleep-dependent memory deficits [31, 32]. In this context, this study aims to untangle the potential of NIR-transcranial treatment on the hippocampal injury produced by paradoxical sleep deprivation (PSD) and age-related SD aggravation. To explore the NIR curable action, we used NIR laser source at a power of 100 mW and wavelength of 830 nm on young and senile Wistar rats. The treatment was employed on 72 h PSD-young (< 2 months) and PSD- senile (> 14 months) rats, and we assessed cognitive function, oxidative stress markers (malondialdegyde, reduced glutathione and superoxide dismutase), and acetylcholine neurotransmitter in the hippocampus. We further evaluated neuronal survival (BDNF and GLP-1 mRNA), apoptotic cell death (Bax and Bcl-2 mRNA), and hippocampal histopathology. To best of our knowledge, this is the first time to evaluate the effectiveness of NIR on the hippocampus of young and senile sleep-deprived rats.

## Materials and Methods

### Animals

Forty-eight male Swiss albino rats (Rattus norvegicus) were used in this study and divided into two groups: 24 rats were 2 -months old (average weight 150 gm), and the rest were 16 -month-old rats (average weight of 350 gm). Animals were obtained from a local breeding house, receiving regular laboratory food and water ad libitum. They were housed 4/cage at 22–24 °C room temperature, humidity (40–60%), and light (12/12 h light/dark cycle). All experimental animals were acclimatized for seven days prior to the experiment initiation. The experiments were done per the Alexandria University guideline for animal care and approval no. (AU. 04 21 10 16 2 01 dated 16 Oct. 2021), in accordance with ICLAS guidelines for the ethical care of experimental animals.

### Experimental Protocol

**Fig. 1:**
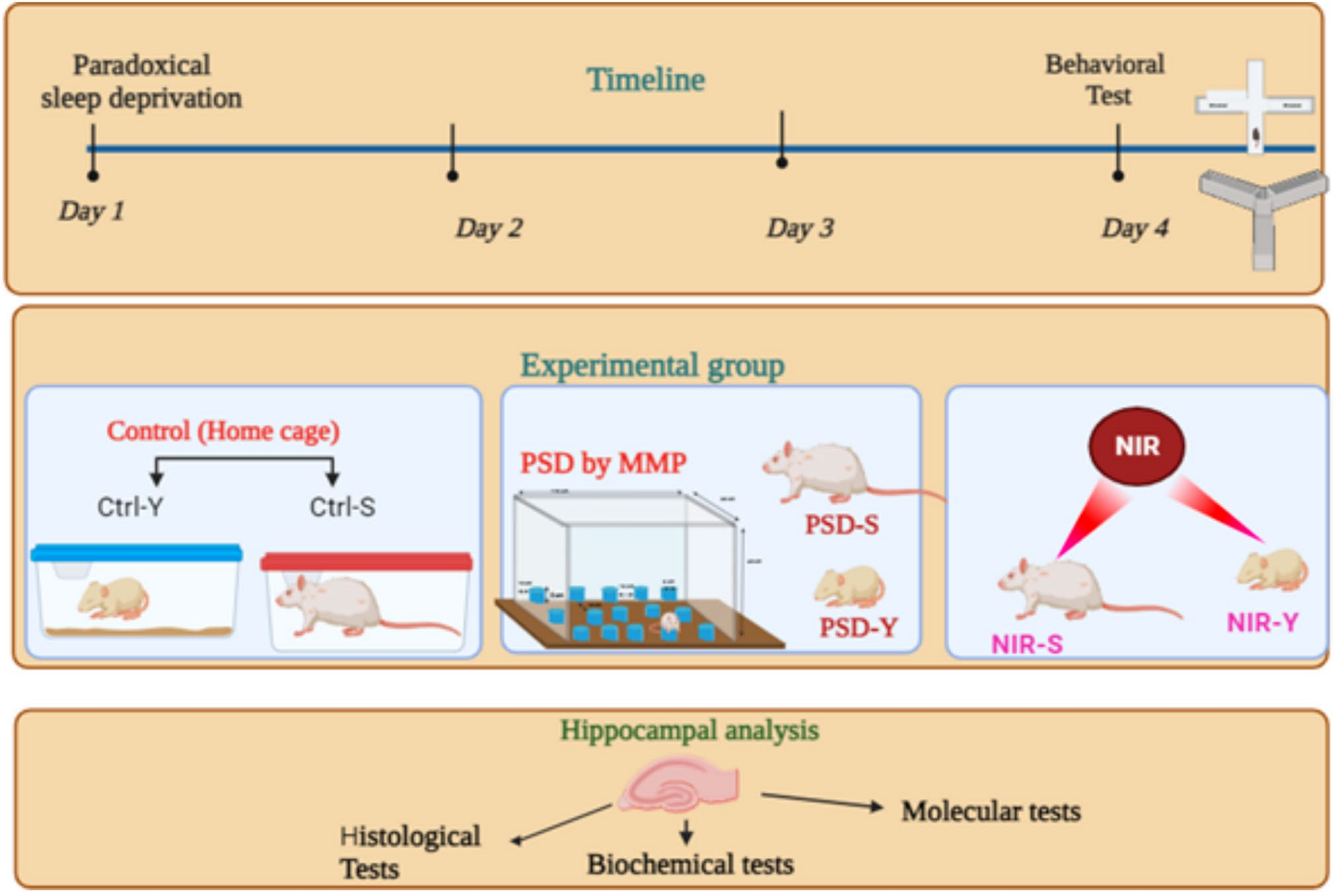
Schematic diagram of experimental procedures. Image created by BioRender.com

Rats were divided into six groups: Control young (Ctrl-Y, n = 8) and control Senile (Ctrl-S, n = 8), who were allowed to stay in their home cage to maintain spontaneous sleep. Paradoxical sleep-deprived young (PSD-Y, n = 8) and Paradoxical sleep-deprived senile (PSD-S, n = 8) who were sleep-deprived for 72 h. NIR laser treatment young (NIR-Y, n = 8) and NIR laser treatment senile (NIR-S, n = 8) who were sleep deprived for 72 h while applying NIR treatment for the same time (Fig. 1).

### Induction of PSD

Induction of REM sleep deprivation was performed in a socially stable environment, where a modified multiple platform (MMP) of the following dimension (110 × 60 × 40 cm^3^) was constructed to facilitate the presence of 15 circular columns (6.5 cm diameter, 8 cm height) [33]. The tank was roughly filled with 7 cm of water, 1 cm below the column flats. Paradoxical sleep-deprived groups were launched into the water, forcing the animals to move to the platform due to water fear; as the subjects slipped into the Paradoxical sleep (PS), the postural tone was lost (atonia phase). Subsequently, the rodent loses control partially or entirely, falling into the water [19, 34] where they were kept for 72 h with full access to food and water.

### Near Infra-red (NIR) Laser Treatment

Treatment groups received NIR laser treatment in parallel to the SD process. The laser treatment was applied on the first day before launching to the MMP apparatus, while on the second and third days, the rats were temporarily removed from the MMP, treated by laser and then reintroduced to the apparatus. In the meantime, the animals were gently held to receive treatment. A diode laser (GaAlAs, Lasotronic Inc., Zug, Switzerland) functioning at 830 nm with an output of 100 mW was employed on six points of shaved heads of the treated animals [35]. The six points were distributed sagitally on each side of the longitudinal commissure and between bregma and lambda. Each point received two min of laser treatment, making the whole session 12 min (Suppl. Table 1).

### Y-Maze (YM) Test

The YM test was used to evaluate short-term working memory. Working memory was measured using the spontaneous alternation method, where the entry number and percentage of alternation were computed according to Kraeuter et al. [36]. After 72 h of REM SD, the animals were removed from MMP and allowed to acclimate in the test room for 1 h. The animals were gently handled, centered in the YM center, and were allowed to roam freely for 8 min and recorded. The plugin MouBeAt from Image-J (http://rsbweb.nih.gov/ij/) was applied to calculate the spontaneous alternation results.

### Elevated Plus Maze (EPM) Test

After 72 h of SD, anxiety-like behavior was assessed using an EPM. Rats were removed from MMP, acclimatized for 1 h prior to the test, and gently placed in the maze’s center. The animals were allowed to roam freely for 5 min [37]. The test was videotaped, and MouBeAt software was used for data acquisition. The number of open arm entries, the time spent in the open arm, the distance covered in the open arm, and the total distance were computed.

### Tissue Sampling

Once all behavior assessments were accomplished, rats were sacrificed. Five animals underwent a rapid decapitation, where the brains were quickly removed, weighed, and separated. Animals brains were dissected to isolate the hippocampus; the left hemispheres were extracted to evaluate biochemical markers, while the right hemispheres were processed for molecular investigation. For the microscopical studies, three rats from each group were perfused via the heart’s left ventricle using 4% paraformaldehyde in 0.1 M phosphate buffer saline (PBS). The whole brains were removed and preserved in 10% formalin for histological analysis.

### Biochemical Assays

The left hippocampi were homogenized (10% w/v) in 4 ml of ice-cold sucrose buffer (0.25 M). The homogenate was then centrifuged at 10,000 ×*g* for 20 min at 4 °C. The supernatant was stored at − 4 °C before biochemical analysis. In order to estimate the activity of hippocampal acetylcholine (ACh), the method of Oswald et al. [38] was used and assayed according to Biovision protocol (Cat No E4454-100) using an ELISA reader at 450 nm. The acetylcholinesterase (AChE) activity was measured using the Kamiya biomedical protocol (Cat No KT-5344) using the ELISA reader at 450 nm [39]. Malondialdehyde (MDA), a metabolite resulting from lipid peroxidation, was measured based on the ability of thiobarbituric acid to react with it, creating a pink color which is estimated at 534 nm using the method described by Ohkawa et al. [40] and the Biodiagnostic protocol (Cat No MD 25 29). The antioxidant activity of reduced glutathione (GSH) was determined according to the biodiagnostic kit protocol (Cat No GR 25 11), where the absorbance was measured at 405 nm [41]. The free radical scavenger superoxide dismutase (SOD) was estimated based on NADH’s ability to cease nitroblue tetrazolium (NitroBT) reduction [42]. The metalloenzyme activity of SOD was assayed at 560 nm using the Biodiagnostic protocol (Cat No SD 25 21).

### Histological Studies

Fixed brains were dehydrated within increasing ethanol concentrations, cleared in xylene, and embedded in paraffin wax as per regular techniques [43]. Paraffin coronal sections were mounted and stained with hematoxylin and eosin to investigate the histological alterations within the different experimental groups.

### Quantitative Real-Time (qRT)-PCR

Total RNA was extracted from the hippocampus after 50 mg of tissue was treated with trizol Invitrogen (Cat No 15,596,026) (ThermoFisher Scientific; Waltham, MA, United States) according to the manufacturer protocol, where the concentration of the extracted product was measured by a NanoDrop® ND–1000 Spectrophotometer (NanoDrop Technologies, United States) to measure the A260/A280 ratio. Following the directions of Thermo Scientific’s Maxima First Strand cDNA Synthesis Kit for RT-qPCR (Cat No K1641), one µg of total RNA was used to create cDNA. Samples were incubated in a Veriti 96-well thermal cycler for 10 min at 25 °C and then 15 min at 50 °C (Applied Biosystems, Applied Biosystems, Foster City, CA). The q RT-PCR was performed using an Mx3005P Real-Time PCR System (Agilent Stratagene, USA) and a Maxima SYBR Green qPCR Master Mix (2X) (Cat. No. K0251), Mix Plus per the manufacturer’s instructions (ThermoFisher Scientific; Waltham, MA, United States). During the PCR cycling procedure, 40 cycles of initial denaturation at 95 °C for 15 s, annealing at 60 °C for 30 s, and extension at 72 °C for 30 s were followed. After converting the expression levels of the target genes to those of the housekeeping gene (GAPDH), the relative fold changes were computed using the 2^−ΔΔCT^ comparative formula [44]. The specific primers generated by Sangon Biotech (Beijing, China) are listed in Suppl. Table 2.

### Statistics

The data underwent statistical computation and were presented as the mean ± standard error mean. The statistical analysis was conducted using Minitab® software version 17. The statistical significance was assessed via analysis of variance (ANOVA). The impact of each age on PSD and treatment was evaluated by implementing a one-way ANOVA, while the interaction of age and NIR treatment on the observed outcomes was assessed using two-way ANOVA. The post-hoc Tukey’s test was conducted per approved statistical procedures. Statistical significance was determined by considering a significance level of p < 0.05.

## Results

### NIR Exerted an Ameliorative Effect on the Working Memory of Young, but not Senile PSD Rats in the YM

**Fig. 2:**
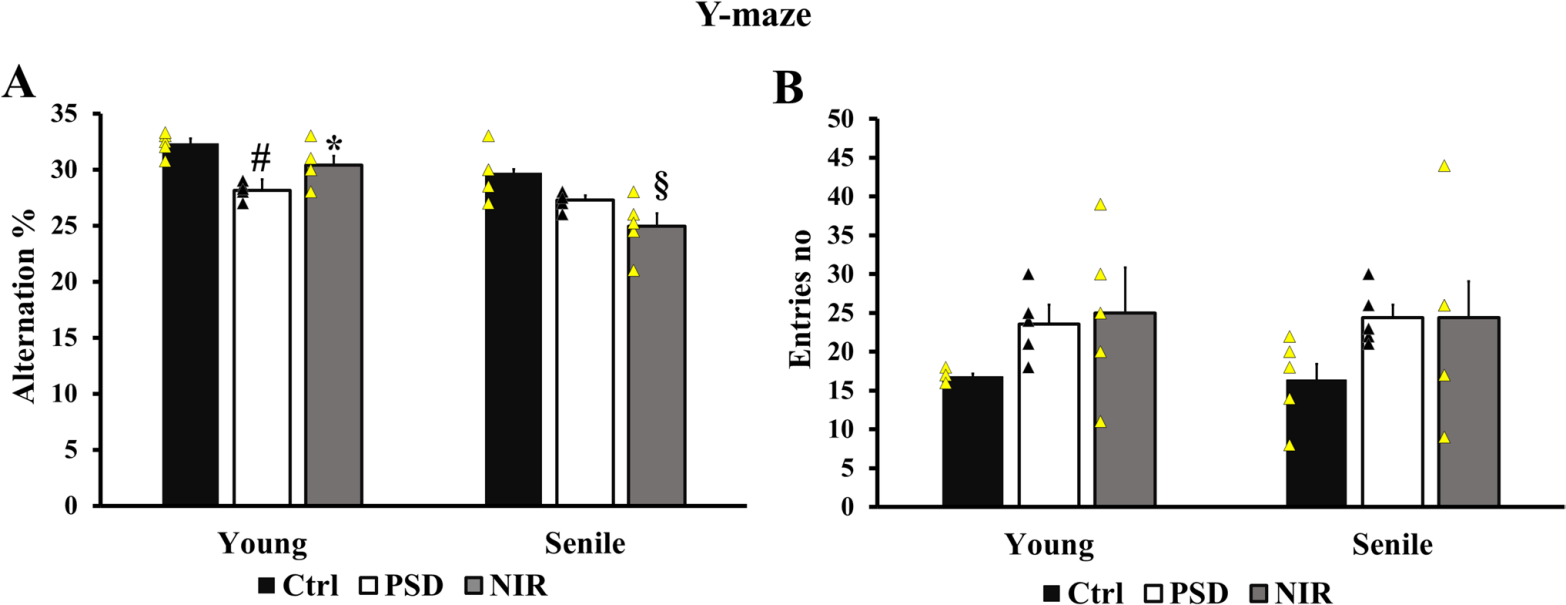
Effect of aging, PSD, and NIR treatment on working memory measured by Y-maze. **A** alternation percentage, and **B** the number of entries made by the animals during the test. Statistically significant means (p-value < 0.05) are given # compared to the respective control (one-way ANOVA/Tukey), * to the respective PSD (one-way ANOVA/Tukey), and § to the corresponding age pattern (two-way ANOVA/Tukey), n = 5/group

The present data (Fig. 2A) showed a significant reduction in the alternation percentage of PSD young animals with a mean value of (28.15% ± 0.3316) in comparison to the control young (32.32% ± 0.44). The NIR-treated group of young rats showed a significant increase in the alternation percentage (30.4% ± 0.812) compared to the PSD young. However, there was a nonsignificant decrease between the results obtained from senile PSD groups (27.3% ± 0.3741) and control senile (29.7% ± 0.994). Also, there was no significant difference between the senile NIR (24.95% ± 1.146), PSD, and control animals. Worthy noted that the alternation percentage was significantly increased in the NIR-Y animals (30.4% ± 0.812) when compared to NIR-S (24.95% ± 1.146) animals (Fig. 2A). Additionally, there was a nonsignificant increase in the number of entries made by PSD-S (24.4 ± 1.63) and PSD-Y (23.6 ± 2.01) compared to their controls. Similarly, there was no significant difference between the entry no. of PSD and NIR-treatment groups in both age groups. Two way ANOVA revealed no significant difference between NIR-Y and NIR-S (Fig. 2B).

### NIR Reduced Anxiety (mania)-Like Effect in Young and Senile PSD Rats as Displayed in the EPM

**Fig. 3:**
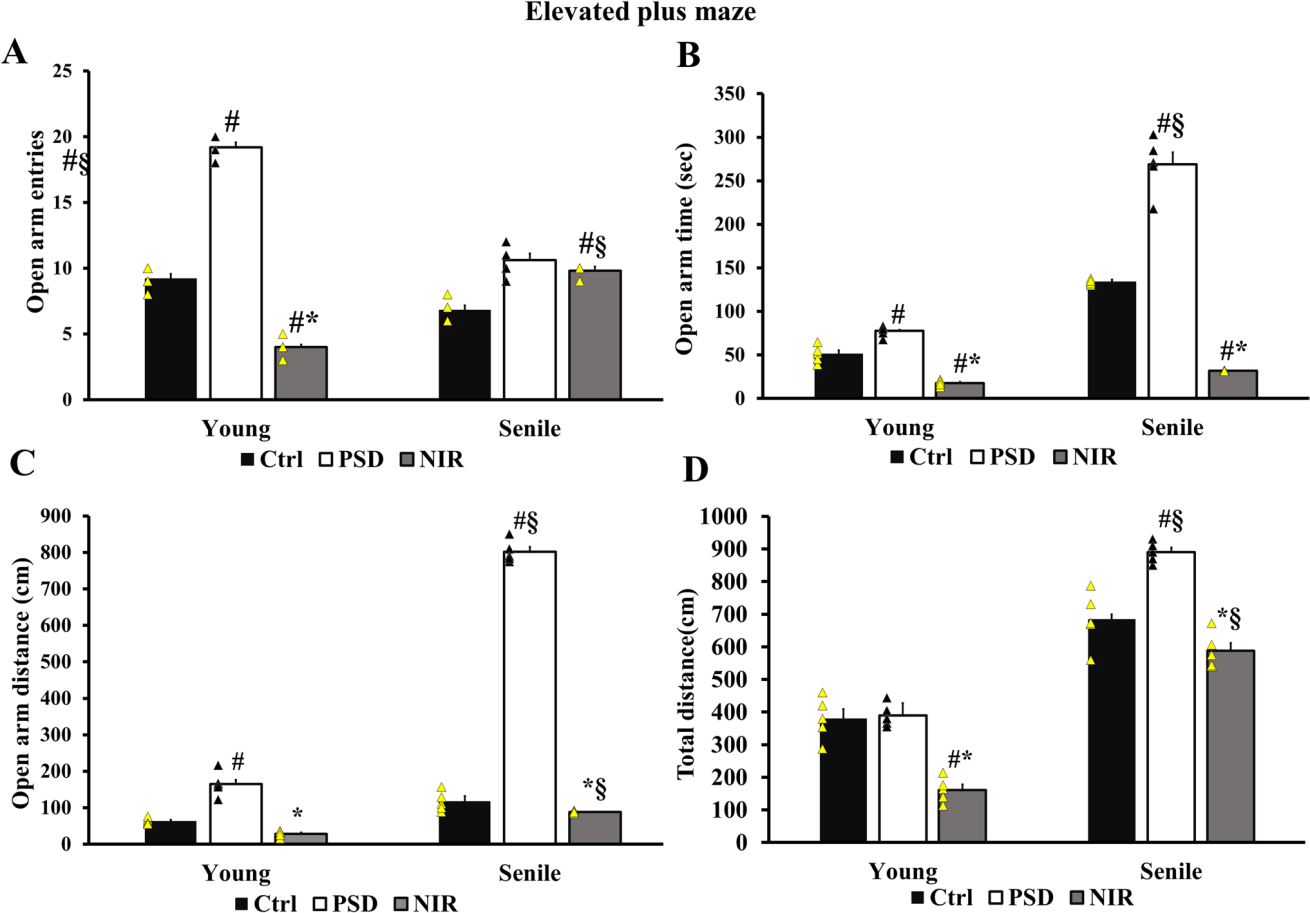
Mania-like behavior assessed by elevated plus maze. **A** open arm entries, **B** total time spent in open arm, **C** total distance covered in open arm, and **D** total distance covered through the whole test. Statistically significant means (p-value < 0.05) are given # compared to the respective control (one-way ANOVA/Tukey), * to the respective PSD (one-way ANOVA/Tukey), and § to the corresponding age pattern (two-way ANOVA/Tukey), n = 5/group

Age and PSD induced a mania-like effect on rodents, where there was a significant increase in the number of open-arm entries made by the PSD-Y (19.2 ± 0.37) compared to Ctrl-Y (9.2 ± 0.37). The NIR significantly reduced the entries number made in the open arm by NIR-Y (4 ± 0.316) compared to Ctrl-Y and PSD-Y. The entries number made by PSD-S significantly increased (10.5 ± 0.5) compared to Ctrl-S (6.8 ± 0.37). NIR-S animal groups performed a number of entries (9.8 ± 0.2) different than those of Ctrl-S (Fig. 3A). It is worth noting that NIR-Y exerted a number of entries less than NIR-S. A significant augmentation in the total time spent in the open arm by PSD-Y (77.6 s ± 2.8) when compared to Ctrl-Y (51.07 s ± 4.466). The time was significantly lowered in the NIR-Y group (17.36 s ± 1.4) compared to the control and PSD groups. Similarly, PSD-S performed more time (268.8 s ± 14.17) in comparison to Ctrl-S (133.8 s ± 1.35). NIR-S spent less time in the open arm (31.70 s ± 0.13) compared to Ctrl-S and PSD-S (Fig. 3B). Two-way ANOVA found no significant difference between NIR-Y and NIR-S. A significant surge in the distance covered in the open arm by PSD-Y (165 cm ± 15.07) in comparison to Ctrl-Y (63.37 cm ± 3.6). NIR significantly decreased the distance covered in the open arm by NIR-Y (28.7 cm ± 3.69) compared to PSD-Y. PSD-S tackled more space (801 cm ± 13.35) than their controls (117.2 cm ± 12.08). At the same time, NIR-S covered a distance of (88.38 cm ± 1.27) less than PSD-S. As expected, NIR-Y had a better performance than NIR-S (Fig. 3C). The total distance by the PSD-Y animal group was insignificant (389 cm ± 15.9) compared to Ctrl-Y (380.17 cm ± 29.4). NIR significantly shrank the total distances made by NIR-Y (161.35 cm ± 17.10) compared to PSD-Y. A significant uprise in the total distance covered by PSD-S (890 cm ± 14.14) compared to Ctrl-S (684 cm ± 37.84) was found. The NIR-S group crossed a shorter distance (587.58 cm ± 24.5) when compared to PSD-S. NIR-S traveled a significantly longer distance than NIR-Y (Fig. 3D). These results showed that NIR counteracts the impulsive and manic behavior induced by PSD in both young and senile animals.

### NIR Reversed the PSD Effect on ACh and AChE in Young and Senile PSD Rats

**Fig. 4:**
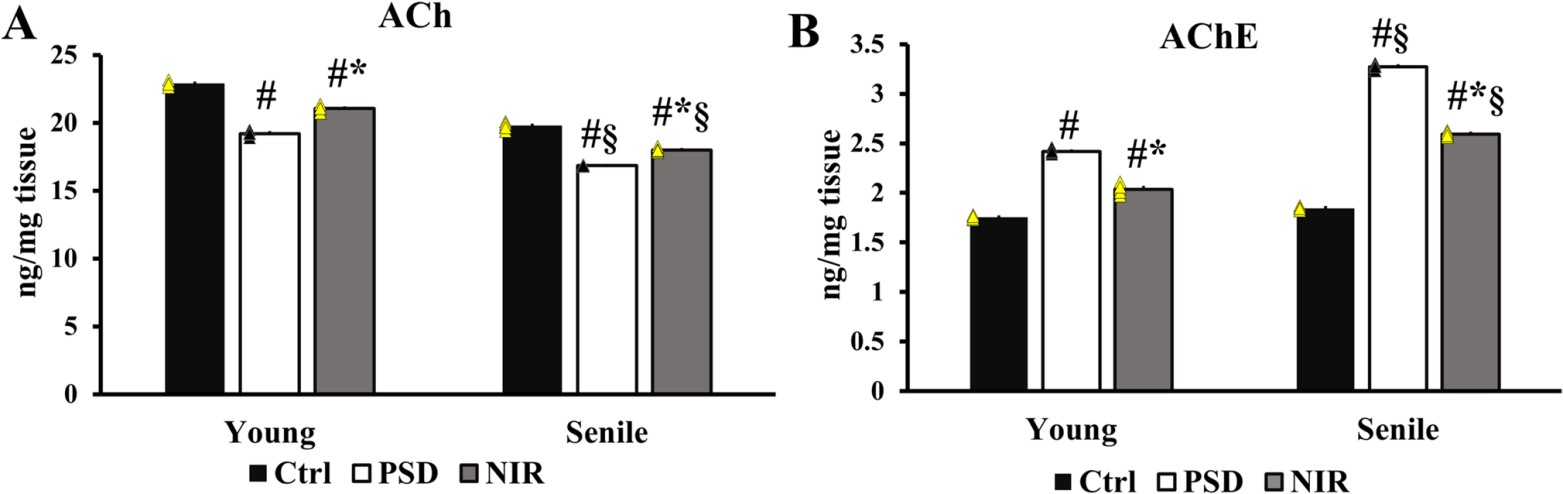
Effect of aging, PSD, and NIR laser treatment on hippocampal neurotransmitter activity. **A** ACh, and **B** AChE. Statistically significant means (p-value < 0.05) are given # compared to the respective control (one-way ANOVA/Tukey), * to the respective PSD (one-way ANOVA/Tukey), and § to the corresponding age pattern (two-way ANOVA/Tukey), n = 5/group

A significant decrease in ACh levels was observed in the hippocampal tissue of PSD-Y (19.19 ± 0.09 ng/mg tissue) in comparison to Ctrl-Y (22.876 ± 0.08 ng/mg tissue). The ACh levels were significantly augmented in NIR-Y (21.05 ± 0.107 ng/mg tissue) compared to the PSD-Y and Ctrl-Y animals. Additionally, the ACh levels of PSD-S (16.852 ± 0.022 ng/mg tissue) were significantly less than those of Ctrl-S (19.758 ± 0.123 ng/mg tissue) and those of NIR-S (18 ± 0.074 ng/mg tissue). Moreover, the age pattern was displayed in the significant rise of the NIR-Y group over the NIR-S group (Fig. 4A). Conversely, there was a significantly increased activity in AChE of the PSD-Y hippocampus (2.41 ± 0.011 ng/mg tissue) in comparison to Ctrl-Y (1.754 ± 0.0087 ng/mg tissue). On the contrary, the AChE levels of the NIR treatment in NIR-Y were significantly reduced (2.0364 ± 0.023 ng/mg tissue) compared to the PSD-Y and Ctrl-Y groups. Concomitantly, PSD-S was significantly augmented (3.27 ± 0.012 ng/mg tissue) compared to both Ctrl-S (1.843 ± 0.0079) and NIR-S (2.5 ± 0.012 ng/mg tissue). An age pattern was shown in the significant decline of the AChE efficiency between NIR-Y and NIR-S (Fig. 4B).

### NIR Improved Hippocampal Redox Status in Young and Senile PSD Rats

**Fig. 5:**
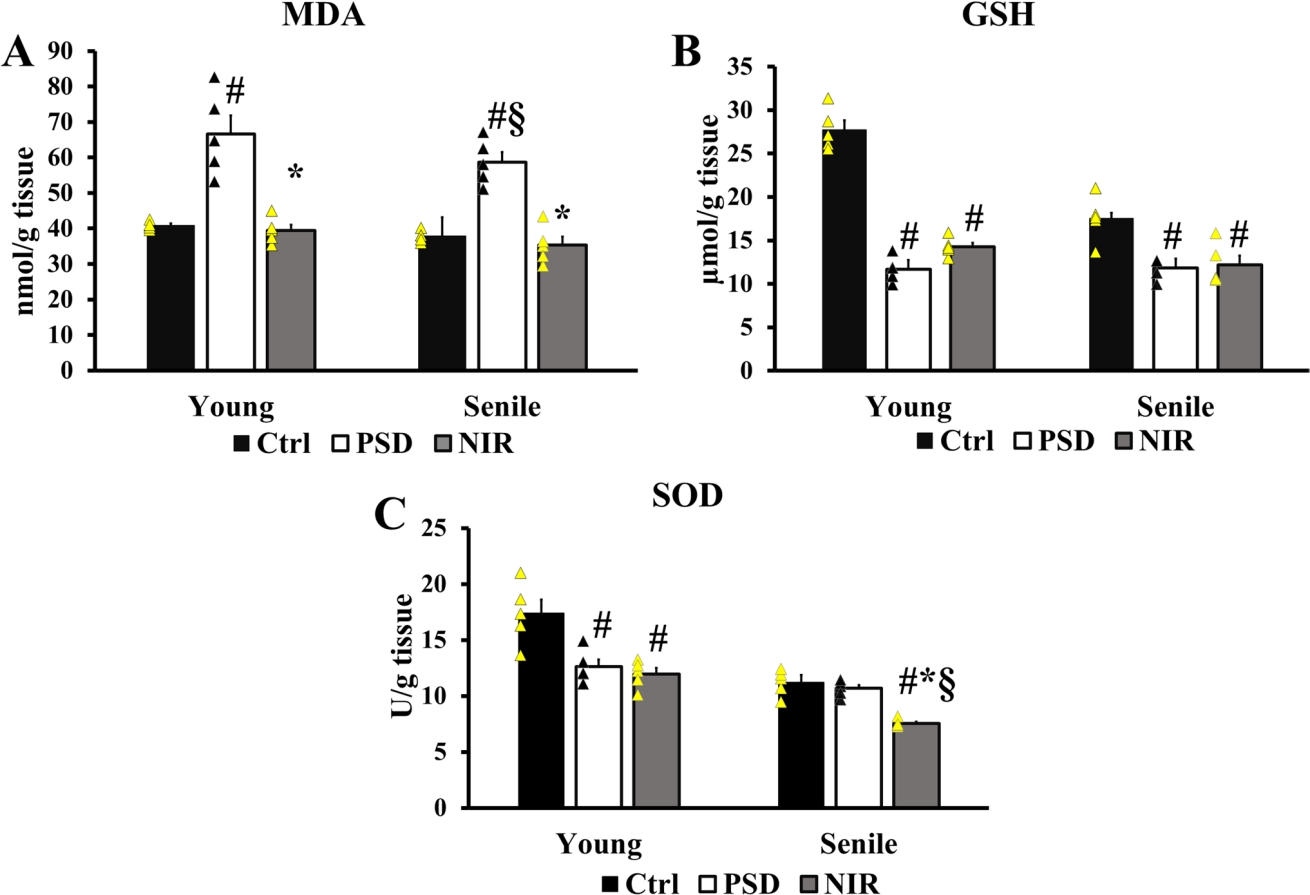
Effect of aging, PSD, and NIR laser treatment on the hippocampal oxidative stress markers. **A** MDA, **B** GSH, and **C** SOD. Statistically significant means (p-value < 0.05) are given # compared to the respective control (one-way ANOVA/Tukey), * to the respective PSD (one-way ANOVA/Tukey), and § to the corresponding age pattern (two-way ANOVA/Tukey), n = 5/group

A significant increase in the levels of hippocampal MDA of PSD-Y (66.64 ± 5.24 nmol/g tissue) in comparison to Ctrl-Y (40.86 ± 0.49 nmol/g tissue). While The NIR-Y treated, groups showed a significant ability to ameliorate the SD effect, where there was a significant shortening in the level of MDA of NIR-Y (39.4 ± 1.62 nmol/g tissue) compared to the PSD-Y group. A significant incline of PSD-S MDA level (58.67 ± 2.8 nmol/g tissue) was noted compared to Ctrl-S (37.86 ± 0.70 nmol/g tissue). Moreover, NIR-S displayed a lower level of MDA (35.339 ± 2.3 nmol/g tissue) compared to PSD-S groups. However, there was no significant difference between the MDA levels of NIR-Y and NIR-S (Fig. 5A). Conversely, there was a significant reduction in the GSH level of the PSD-Y groups (11.66 ± 0.64 mg/g tissue) compared to their Ctrl-Y (27.75.834 ± 1.04 mg/g tissue). GSH levels of NIR-Y (14.269 ± 0.47 mg/g tissue) were insignificantly elevated compared to the PSD-Y group and significantly different from Ctrl-Y. Additionally, a significant decline in the GSH activity of PSD senile animals (11.834 ± 0.54 mg/g tissue) compared to Ctrl-S (17.517 ± 1.70 mg/g tissue) was noted. An insignificant surge of NIR-S GSH (12.18 ± 1.05 mg/g tissue) compared to PSD-S and a significant difference to Ctrl-S was found. GSH amounts were unrelated to the age pattern displayed in this study (Fig. 5B).

The SOD activity of PSD-Y groups significantly declined (12.60 ± 0.65 U/g tissue) compared to Ctrl-Y (17.39 ± 1.22 U/g tissue). Additionally, the SOD levels of NIR-Y, treated group (11.223 ± 1.22 U/g tissue) insignificantly differed from PSD-Y. However, NIR treatment showed a significantly decreased activity than the Ctrl-Y group. There was an insignificant limitation of SOD activity when comparing the PSD-S (10.67 ± 0.31 U/g tissue) to the control senile animals Ctrl-S (11.223 ± 1.22 U/g tissue), while the senile NIR group NIR-S (7.55 ± 0.155 U/g tissue) was significantly different from Ctrl-S, PSD-S. SOD level in the NIR-Y animals was significantly higher than those of NIR-S (Fig. 5C).

### NIR Protected Against PSD-induced Neuronal Damage in Young, but not Senile PSD Rats

**Fig. 6:**
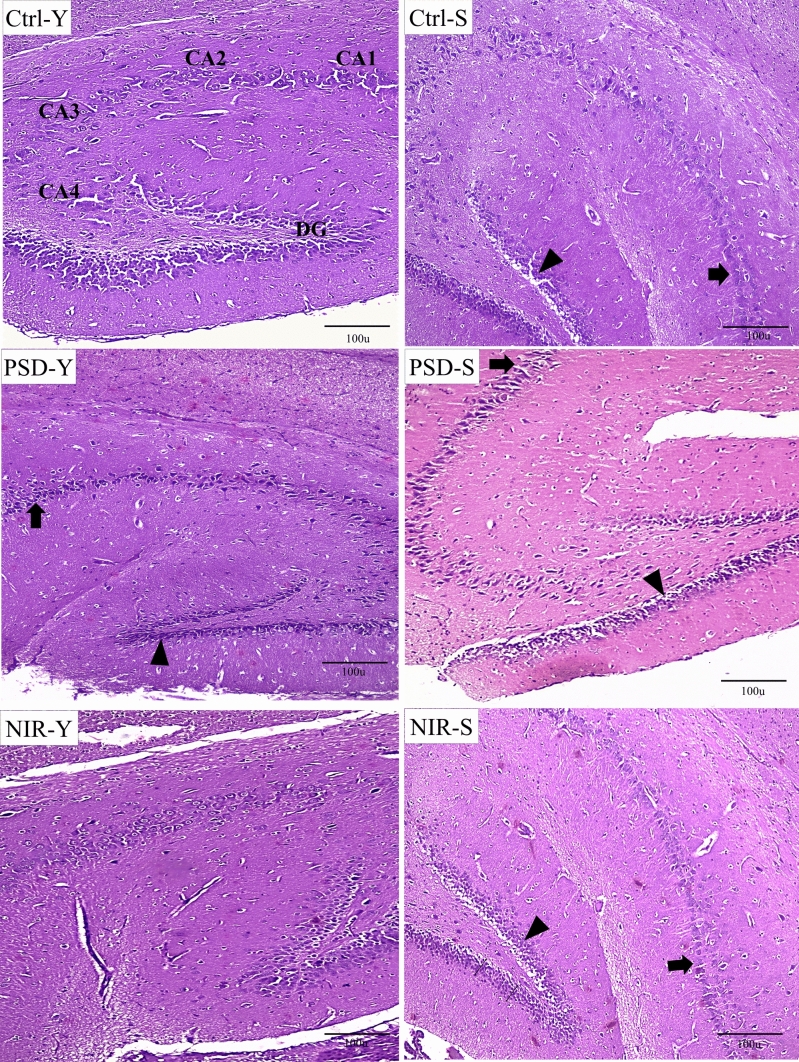
Representative photomicrographs of hippocampal neuronal morphology of the study groups. CA1: Cornu Ammonis field 1, CA2: Cornu Ammonis field 2, CA3: Cornu Ammonis field 3, CA4: Cornu Ammonis field 4, DG: Dentate Gyrus. Observe neuronal lesions in CA1 pyramidal layer (arrows) and DG granular layer (arrowheads). It can be seen that NIR-Y fully restored CA1 and DG cellularity while NIR-S still exhibited signs of tissue damage and neuronal loss. H&E stain. Sacle bar: 100 μm

Histological observations of the rat hippocampus are shown in Fig. 6. We focused on CA1 and DG of the hippocampus because of their documented role in SD-related learning and memory dysfunctions [10–12]. Ctrl-Y animals showed normal histoarchitecture of CA1 and DG subregions of the hippocampus with no evidence of apoptosis or cerebral edema. However, PSD induced pyknotic nuclei (apoptosis) with halos and reduced thickness of both CA1 and DG areas in the case of PSD-Y animals. NIR applied to PSD-Y rats could effectively recover the intrigity of CA1 and DG neurons with minimal disruption to the hippocampus. In senile control (Ctrl-S) animals, we observed scattered apoptic cells and hydropic degeneration (i.e., cell swelling) in CA1 and DG. PSD-S animals also depicted pyknosis and marked hydropic damage of the CA1 and DG neuronal cells. Nevertheless, the deformed CA1 and DG neurons were still found in the NIR-S animals.

### NIR Changed mRNA Expression Levels of Some Neuronal Survival (BDNF and GLP-1)- and Apoptotic (Bax and Bcl-2)-Reated Genes in Young and Senile PSD Rats

**Fig. 7:**
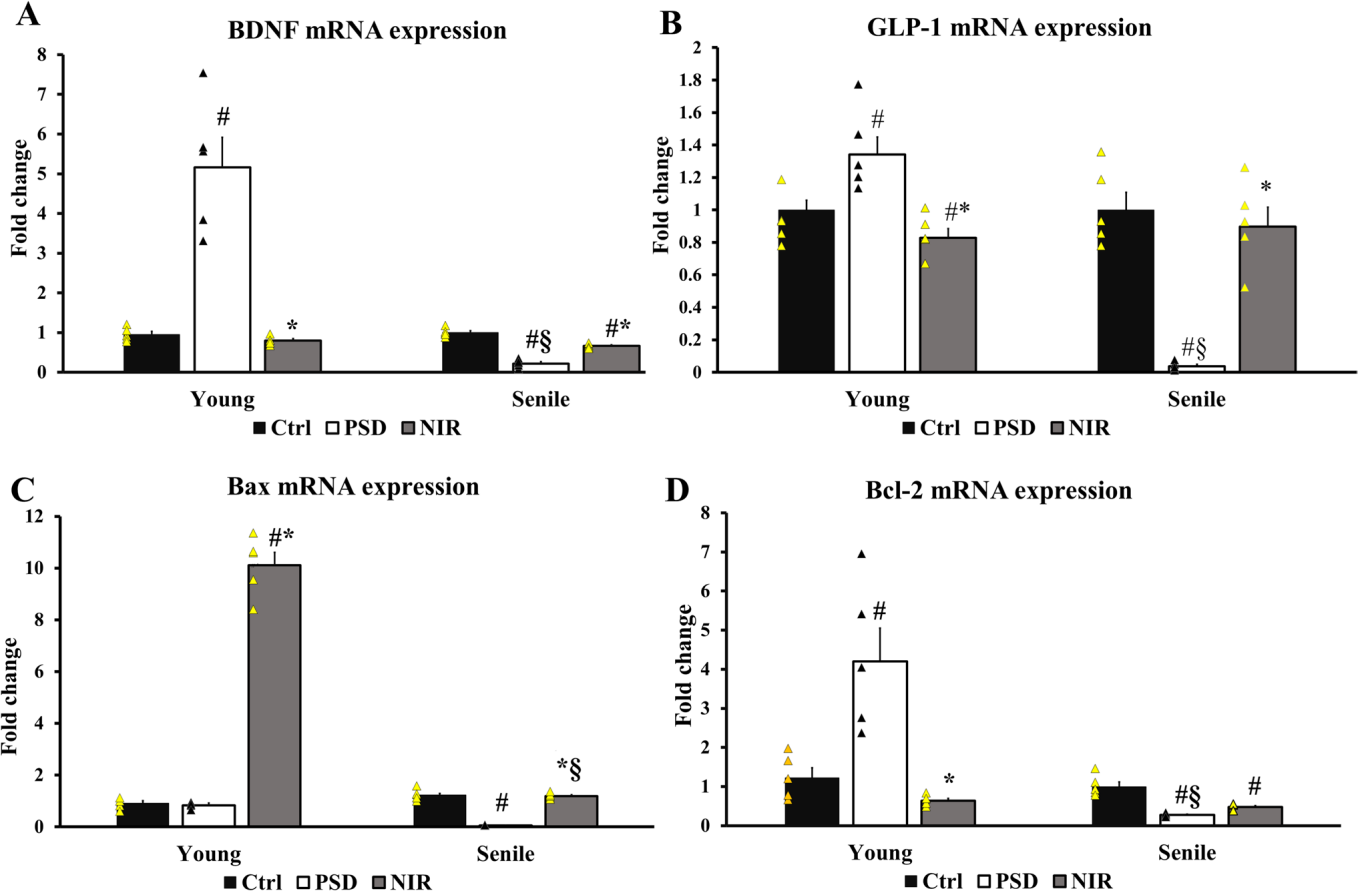
Relative gene expression. **A** BDNF, **B** GLP-1, **C** Bax, and **D** Bcl-2. Control, PSD, NIR-treatment of the young and senile hippocampus. Statistically significant means (p-value < 0.05) were given # compared to the respective control (one-way ANOVA/Tukey), * to the respective PSD (one-way ANOVA/Tukey), and § to the corresponding age pattern (two-way ANOVA/Tukey), n = 5/group

BDNF expression showed a significant increase in the case of PSD-Y (5.16 ± 0.75) in comparison to control young Ctrl-Y (Fig. 7A). NIR treatment significantly reduced BDNF levels of NIR-Y (0.79 ± 0.04) while having no significant difference with the Ctrl-Y group. A significant reduction in BDNF expression level of PSD-S (0.22 ± 0.04) compared to Ctrl-S, in addition to a significant elevation of BDNF expression of NIR-S (0.66 ± 0.02) compared to the PSD-S group. Moreover, no significant difference was noted in the expression of BDNF between NIR-Y and NIR-S. Meanwhile, GLP-1 showed a significant expansion in its expression in the case of PSD-Y (1.3 ± 0 0.119) in comparison to control young Ctrl-Y (Fig. 7B). However, after the NIR treatment, GLP-1 was significantly down-regulated in the NIR-Y (0.82 ± 0.05) group when compared to the PSD-Y group. Within the aged animal groups, a significant decline in GLP-1 expression was noticed in PSD-S (0.037 ± 0.01) compared to Ctrl-S. NIR treatment was able to significantly elevate GLP-1 in the case of NIR-S (0.89 ± 0.12) compared to the PSD-S groups. Our results indicated no significant differences in the GLP-1 expression between NIR-Y and NIR-S. Of note, the present data showed that young age could trigger a protective action against SD by elevating BDNF and GLP-1, which was not the case with elder rats.

We observed an insignificant decrease in the PSD-Y Bax expression (0.663 ± 0.04) compared to Ctrl-Y (Fig. 7C). Surprisingly, NIR elevated Bax expression of NIR-Y (10.11 ± 0.643) compared to PSD-Y and Ctrl-Y. Moreover, a significant decline in Bax expression (0.051 ± 0.004) of PSD-S compared to Ctrl-S was found. NIR treatment was able to restore the baseline in the Bax expression (0.961 ± 0.05) of the NIR-S group compared to PSD-S. A significant elevation in Bax levels of NIR-Y was evident compared to NIR-S. As shown in Fig. 7D, a significant increase in the expression of Bcl-2 (4.20 ± 0.84) of PSD-Y was noticed compared to Ctrl-Y. NIR significantly restored the regular expression of Bcl-2 (0.64 ± 0.05) compared to Ctrl-Y and PSD-Y. Additionally, there was a significant reduction in Bcl-2 expression (0.27 ± 0.01) of PSD-S compared to Ctrl-S. NIR treatment attenuated changes in Bcl-2 expression in the NIR-S group, which insignificantly up-regulated Bcl-2 (0.480 ± 0.03) compared to PSD-S. Nevertheless, no significant difference in the expression of NIR-Y and NIR-S was found.

## Discussion

Epidemiological studies have linked sleep loss to the development of pathological neurodegenerative ailment [45] and a decline in cognitive function [46]. Insomnia (i.e., difficulty in falling asleep) has been described to increase among elders. It is still debatable whether sleeplessness is linked to aging or functions as a particular stressor that triggers pathological processes like neurodegeneration [47]. That said, digging into the mechanisms of aging and SD at various organizational levels and creating a solid strategy for guaranteeing healthy aging are becoming more crucial in light of the global population’s steadily rising life expectancy, especially with limited sleep. The key question of this study was to assess how far the NIR-transcranial treatment can improve cognitive impairment and hippocampal neuronal damage induced by the putative disruptive nature of PSD, and the consequences of age-related SD adversity were also investigated.

It is eminent that chronic SD has a detrimental effect on memory retention, mood, and other cognitive performances, learning capability, and memory retention as measured by different tests. Nevertheless, limited studies have investigated the cognitive disruption resulting from PSD in senile experimental animals. The effect of PSD on the tested animals in the current study has shown an age-dependency pattern. Spatial working memory was measured using YM, and the results showed a significant reduction in the alternation percent made by young PSD compared to their control rats. However, senile PSD animals revealed a nonsignificant decrease in alternation percent compared to senile control. Lima et al. [48] showed that 48 and 72 h of PSD have decreased mice working memory alternation, where 48 h had the most adverse effect. Similar to the present findings, age-depentent memory impairments were also observed for short-term sleep restriction [49]. Furthermore, it was reported that aged animals may be hyporesponsive to severe psychological stress at the expense of their physiological capacity to adapt [50]. On the other hand, our results showed that both senile and young PSD rats displayed an increased level of anxiety as indicated by the EPM. These results are consistent with a previously published report using 72 h of REM-depleted animals [51]. Also, a study investigated the effect of PSD and Formalin on pain and anxiety, and found that PSD increased the time spent in the open arm compared to formalin [52]. Sleep loss is commonly used to create mania and bipolar depression animal models, and it is clinically proven to exacerbate psychological symptoms in elderly patients [53].

For the first time in the current study, the age pattern was considered while evaluating the NIR ability to modulate the SD effect. NIR enhanced spatial working memory tested by YM in NIR-Y but did not affect those of the senile. While, we found that the NIR treatment ameliorated anxiety-like and impulsive behavior in both young and senile rats. Although limited studies were done to investigate NIR ability to mitigate psychological disorders resulting from PSD, clinical and animal studies have referred to the antidepressive power of NIR. Previously, NIR (80 mW) was shown to mitigate depression and anxiety-like behavior in rats-induced by by reserpine, and the irradiated depressed animals reduced immobility and increased their swimming time in the Forced swimming test [54]. Furthermore, lithium-treated bipolar patients were exposed to 830 nm of t-PBM radiation to eradicate their symptoms, and anhedonia decreased due to radiation therapy, followed by improved anxiety, sleep, irritability, and impulsivity [55]. The anti-anxiety and anti-depressive effects of NIR PMB is probably linked to increasing serotonin and decreasing nitric oxide levels in the prefrontal cotex and hippocampus areas [56].

Whether the reason is PSD or aging, the resulting cognitive impairment could be attributed to altered hippocampal ACh and cellular redox activity. Several studies have mentioned ACh/AChE imbalance to alter hippocampal function, resulting in memory impairment related to various neurodegenerative disorders such as Alzheimer’s [57]. In addition, it was found that aged rats (22 month-old) whose prefrontal cortex has less ACh failed to solve proactive interference odor discrimination tasks [58]. Moreover, the altered hippocampal redox state produced by SD [59] or aging [60] could be the initiator culprit of the disruptive memory consolidation process. Consequently, the hippocampal lipid peroxidation and passive avoidance retention deficit could be avoided by antioxidants agents such as melatonin and vitamin E [61]. Our results align with previous studies, in which 72 h sleep-deprived mice showed a reduced cholinergic neurotransmitter ACh, elevated AChE, and reduced learning ability [62, 63]. Moreover, patients with insomnia and cerebral infarction had lower ACh levels in their plasma [64]. Our data found an extensive increase in lipid peroxidation, indicating free radical activity, accompanied by a reduction of GSH (in young and senile PSD) and SOD (in young PSD). The abovementioned effect is consistent with a study demonstrating that sleep-deprived rats had higher levels of MDA with a reduction in GSH and an increase of AchE activity [65]. ACh is not only known for its role in hippocampal-dependent memory consolidation, but it is also known for its anti-oxidative stress ability [66, 67]. It was found that ACh effectively attenuated the reduction in SOD activity brought on H9c2 cells by hypoxia/reoxygenation (H/R) [68]. Furthermore, a study also noticed the interchangeable relationship between oxidative stress and cholinergic neurons, which found that administering the ACh antagonist scopolamine increased AChE, MDA, and impaired memory learning [69]. In fact, the local release of ACh-induced glutamate release through activation of nAChR occurs in many parts of the CNS related to memory formation [70]. Infrared light proved to be able to induce excess efflux of glutamate by stimulating glutamatergic nerve endings [71]. Our data demonstated that NIR contributed differentially to increase ACh and decrease AChE in NIR-Y than NIR-S. The PBM therapy applied in this study also resulted in mitigation of the SD oxidative effect (MDA levels) in young and senile rats. In a recent report, researchers found that combined PBM therapy and methylene Blue, a reduction-oxidation agent, could mitigate learning impairment and oxidative stress produced by unpredictable chronic mild stress [72].

The findings of the present histological analysis align with a study that showed neuronal autophagy and apoptosis in the hippocampus of mice after SD for 48 h [73]. Moreover, excess apoptosis can be manifested through excessive intracellular ROS, and promoting mitochondrial fission [74]. Here, the photomicrograph of NIR-treated animals (NIR-Y) showed intact nuclei of the pyramidal layer similar to Ctrl-Y, indicating complete improvement with no evidence of apoptosis or edema compared to PSD-Y. In addition, the transcranial treatment of NIR-S animals has decreased the rate of apoptosis and edema compared to PSD-S groups. The activity of NIR is well documented to release vasodilator compounds [25]. The vasodilation impact of NIR improves cerebral blood flow [75], consequently decreasing ROS production, allowing oxygen to regain functionality, and increasing oxygen consumption, mitochondrial function, and cerebral oxygenation [76]. This can explain the improved redox state mentioned in this study.

Sleep loss is fundamental to gene expression alteration [77]. Moreover, an age-dependent pattern of various gene expressions was noticed in the present data. Senile PSD showed a reduction in BDNF, a neurotrophic factor that participates in neuronal plasticity. While, young PSD animals displayed overexpression in the BDNF levels. Ma et al. [78] demonstrated that 6 h of acute SD impaired short and long-term memory measured by fear memory performance, and this was accompanied by a compensatory increase in BDNF protein expression and activation of TrkB/PLCγ1 signaling in the basal forebrain. Also, Gorgulu et al. [79] have shown that acute SD could increase serum BDNF levels in healthy subjects. However, it has been described that hippocampal BDNF levels decreased in aged rats as compared to young adults [80]. Li et al. [81] found that low levels of AChE increased BDNF and nerve growth factor (NGF). Thus, in senile PSD animals, the downregulation of BDNF mRNA expression could be correlated to the elevation of AChE. Moreover, the BDNF reduction in PSD-S can result in apoptosis and poor cognitive ability [20]. PBM ability involves NIR absorption through chromophore compounds like cytochrome and transient receptor protein family (TRP) [32], which are activated by various stimuli and have versatile cellular roles [82]. TRPC mediates BDNF, sustaining membrane depolarization and increasing dendritic spine density [83]. BDNF TRPC/Ca^+2−^dependent activity promotes embryonic cell line survival and increases ACh and ACh transferase levels [84]. The present data proved that NIR could restore BDNF in both young and senile rats to near normal levels. Previous report found that, by activating the extracellular signal-regulated kinase ERK and CREB signaling pathways, PBMT decreased hippocampal cell line (HT-22) mortality caused by oxidative stress and enhanced BDNF expression. The levels of phosphorylated ERK and CREB, which were reduced by oxidative stress, as well as the production of the antioxidant enzyme SOD, were also enhanced by PBMT in hippocampus organotypic slices [85].

We demonstrated a marked reduction in GLP-1 in senile PSD animals, along with GLP-1 up-regulation in young PSD. These results are in accordance with a study showing that acute SD declined postprandial GLP-1 in healthy men [86]. Another study has found that reduced fasting GLP-1, GIP, and glucose-stimulated GLP-1 levels are linked to aging [87]. Futher, it was shown that GLP1 agonists may provide an alternative metabolic approach for cognitive dysfunction [88]. For instance, the GLP-1 agonist reduced behavior despair resulting from ovariectomy through preserving BDNF, and enhancing cellular defense against ROS [89]. The present data proved that NIR application markedly increased GLP-1 in senile treated animals. The restoration of GLP-1 expression by NIR can protect against oxidative stress and neuroinflammation, increase synaptic plasticity, up-regulate BDNF, decrease apoptosis signaling molecules and rescue EPM performance [90, 91].

This study indicated that Bcl-2 levels had a significant decrease in PSD-S and a significant rise in PSD-Y. Young PSD did not show a substantial increase in Bax expression, whereas senile PSD showed a considerable decrease in Bax levels. In agreement with our results, Montes-Rodríguez et al. [92] noted that 24 h total SD and 24 h sleep rebound caused a decline in hippocampus Bax level and an increase in Bcl-2 level. It is known that a cell’s response to a pro-apoptotic factor may depend on the balance between pro- and anti-apoptotic genes [93]. However, our data are in conflicat with de Souza et al. [17] who found that chronic SD increased Bax levels and did not affect Bcl-2 levels in young and old animals. These discrepancies could be attributed to the fact that these authors investigated the effect of chronic sleep restriction after 21 days, which was not the case in our study. What is more, the increased BDNF in young PSD animals can contribute to the elevated Bcl-2. BDNF exhibited antiapoptotic ability in neurons during ischemic conditions through neutralization of pro-apoptotic protein Bax and counter-regulation of Bcl-2 protein [94]. GLP-1 upregulation could also explain the enhanced Bcl-2 levels in PSD-Y. In addition, GLP-1 neuroprotective effects in animal models and tissue cultures are evident through reducing caspase-3 activity, and down-regulating pro-apoptotic Bax [95]. In the present study, our data showed that NIR caused an unexpected increase in Bax mRNA levels (in young and senile PSD rats), while Bcl-2 expression decreased (in young PSD rats). The mechanism(s) of this effect requires further investigations at the protein levels. Zhang et al. [96] showed that low-power laser irradiation could prevent Bax translocation to mitochondria and caspasse-3 activation; therefore, it could inhibit apoptosis.

## Conclusion

This study explored the effect of NIR against SD and aging-related cognitive impairment. Results showed an age-dependent pattern of working memory dysfunction and mania-like behaviors in PSD rats, which could be attributed to cholinergic dysregulation and altered cellular redox. Age significantly impacted molecular compensatory machinery in young sleep-deprived animals, affecting neurotrophic factor BDNF, neuron survival factor GLP-1, and apoptotic Bax and Bcl-2 genes. The neuroprotective ability of NIR has been observed to counteract the consequences of aging and SD, specifically by mitigating cholinergic dysfunction and neutralizing ROS. Also, NIR enhanced the mRNA expressions of BDNF and GLP-1 (with antiapoptotic actions) in senile rats. Future research is needed to provide a comprehensive understanding of the molecular factors (e.g., glial markers) underlying the therapeutic effects of varying doses, wavelengths of NIR light, and specific characteristics of SD.

### Supplementary Information

Below is the link to the electronic supplementary material.

Supplementary material 1 (DOCX 12.7 kb)

Supplementary material 2 (DOCX 13.6 kb)

## Data Availability

The datasets generated during and/or analysed during the current study are available from the corresponding author on reasonable request.
