# Supplementary material for: Transcranial Irradiation Mitigates Paradoxical Sleep Deprivation Effect in an Age-Dependent Manner: Role of BDNF and GLP-1
Source: Neurochem Res. 2023 Dec 20;49(4):919–34. doi: 10.1007/s11064-023-04071-y (PMC10902205; doi:10.1007/s11064-023-04071-y)
Supplement: Supplementary file 1 — Supplementary material 1 (DOCX 12.7 kb) [file 11064_2023_4071_MOESM1_ESM.docx]

**Table (1). Laser parameters**

| Parameter | Value |
| --- | --- |
| Repetition | 12 min/day for three days |
| Power | 100 mW |
| Spot size | d = 1 cm, A = 0.785 cm2 |
| Irradiance | 127.4 mW/cm2 |
| Fluence | 15.28 J/c |
| Wavelength | 830 nm |
| Energy/point | 11.99 J |
| Total energy/ points (one session) | 71.96 J |
| Mode | CW. |
