# Supplementary material for: Transcranial Irradiation Mitigates Paradoxical Sleep Deprivation Effect in an Age-Dependent Manner: Role of BDNF and GLP-1
Source: Neurochem Res. 2023 Dec 20;49(4):919–34. doi: 10.1007/s11064-023-04071-y (PMC10902205; doi:10.1007/s11064-023-04071-y)
Supplement: Supplementary file 2 — Supplementary material 2 (DOCX 13.6 kb) [file 11064_2023_4071_MOESM2_ESM.docx]

**Table (2). Primers designed for qRT-PCR**

|  | Forward primer | Reverse primer | Accession number |
| --- | --- | --- | --- |
| BDNF | GAAAGTCCCGGTATCAAAAG | CGCCAGCCAATTCTCTTTTTG | NM_001270630.1 |
| GLP-1 | CACCTCCTCTCAGCTCAGTC | CGTTCTCCTCCGTGTCTTGA | NM_012707.3 |
| BCL-2 | GACTGAGTACCTGAACCGGCATC | CTGAGCAGCGTCTTCAGAGACA | NM_016993.2 |
| BAX | CGAATTGGCGATGAACTGGA | CAAACATGTCAGCTGCCACAC | NM_017059.2 |
| GAPDH | GGCACAGTCAAGGCTGAGAATG | ATGGTGGTGAAGACGCCAGTA | NM_017008.4 |
